# Supplementary material for: A meta-interactive neural network for solving time-varying quadratic programming problems
Source: Nat Commun. 2025 Nov 21;16:10284. doi: 10.1038/s41467-025-65192-2 (PMC12638829; doi:10.1038/s41467-025-65192-2)
Supplement: Supplementary file 1 — Supporting Information [file 41467_2025_65192_MOESM1_ESM.pdf]

Supplementary Information

"A Meta-Interactive Neural Network for Solving Time-Varying Quadratic Programming Problems"

Zhijun Zhang[1,2,3,4,5,6,7,8,9,10*†], Xiangliang Sun[1*†], Yiqi Liu[1], Yamei Luo[11]

[1*]School of Automation Science and Engineering, South China University of Technology, Guangzhou, 510640, Guangdong, China.

[2]Key Library of Autonomous Systems and Network Control, Ministry of Education, School of Automation Science and Engineering, South China University of Technology, Guangzhou, 510640, Guangdong, China.

[3]Guangdong University Key Laboratory of Large-Model Embodied-Intelligent Humanoid Robot, Guangzhou, 510640, Guangdong, China.

[4]Institute for Super Robotics (Huangpu), Guangzhou, 510555, Guangdong, China.

[5]Nanchang University, Nanchang, 410205, Jiangxi, China.

[6]College of Computer Science and Engineering, Jishou University Jishou, 416000, Hunan, China.

[7]Guangdong Artificial Intelligence and Digital Economy Laboratory (Pazhou Lab), Guangzhou, 510335, Guangdong, China.

[8]School of Electronical Engineering, Shaanxi University of Technology, Hanzhong, 723001, Shaanxi, China.

[9]School of Information Science and Engineering, Changsha Normal University, Changsha, 410100, Hunan, China.

[10]Institute of Artificial Intelligence and Automation, Guangdong University of Petrochemical Technology, Maoming, 525000, Guangdong, China.

[11]Department of Mechanical and Automation Engineering, The Chinese University of Hongkong, Hong Kong, 999077, China.

[*]Corresponding author(s). E-mail(s): auzjzhang@scut.edu.cn (Zhijun Zhang); sxl245578@163.com (Xiangliang Sun);

[†]These authors contributed equally to this work.

Supplementary Note 1

MINN solves Sylvester equation

Consider the smooth time-varying Sylvester equation in ref. [1], [2] as

$$\bar{\mathbf{A}}(t)\mathbf{X}(t) - \mathbf{X}(t)\bar{\mathbf{B}}(t) + \bar{\mathbf{C}}(t) = 0, t \in [0, +\infty) \qquad \text{(Supplementary Equation 1)}$$

where t denotes time, $\bar{\mathbf{A}} \in \mathbb{R}^{m\times m}$, $\bar{\mathbf{B}} \in \mathbb{R}^{n\times n}$ and $\bar{\mathbf{C}} \in \mathbb{R}^{m\times n}$ are time-varying smooth coefficient matrices. Without loss of generality, the $\dot{\bar{\mathbf{A}}}$, $\dot{\bar{\mathbf{B}}}$, $\dot{\bar{\mathbf{C}}}$ are the derivative of $\bar{\mathbf{A}}$, $\bar{\mathbf{B}}$, $\bar{\mathbf{C}}$ with respect to time. Suppose that the unknown matrix $\mathbf{X} \in \mathbb{R}^{m\times n}$ exists, and we are trying to find the unique solution $\mathbf{X}^*(t)$ to the Sylvester equation, such that the Sylvester equation holds true. To obtain the unique solution to Sylvester equation (1), a matrix-type error function is defined as

$$\boldsymbol{\varepsilon}(t) = \bar{\mathbf{A}}(t)\mathbf{X}(t) - \mathbf{X}(t)\bar{\mathbf{B}}(t) + \bar{\mathbf{C}}(t) \qquad \text{(Supplementary Equation 2)}$$

According to MINN's dynamic design method, the error convergence formula is constructed as

$$\dot{\boldsymbol{\varepsilon}}(t) = -\tau(t)\boldsymbol{\varepsilon}(t) + \boldsymbol{\alpha}\mathbf{F}(\boldsymbol{\varepsilon}(t)) \qquad \text{(Supplementary Equation 3)}$$

where $\boldsymbol{\alpha}$ is a parameter matrix with appropriate dimensions that satisfies the requirements of Theorem 1. Based on the above design formula, the implicit dynamic equation for solving the Sylvester equation under MINN can be obtained as

$$\bar{\mathbf{A}}(t)\dot{\mathbf{X}}(t) - \dot{\mathbf{X}}(t)\bar{\mathbf{B}}(t) = -\dot{\bar{\mathbf{A}}}(t) + \mathbf{X}(t)\dot{\bar{\mathbf{B}}}(t) - \dot{\bar{\mathbf{C}}}(t) - \tau(t)\mathbf{F}(\bar{\mathbf{A}}(t)\mathbf{X}(t) - \mathbf{X}(t)\bar{\mathbf{B}}(t) + \bar{\mathbf{C}}(t)) + \boldsymbol{\alpha}\mathbf{F}(\boldsymbol{\varepsilon}(t))$$

(Supplementary Equation 4)

(Supplementary Equation 4) has provided all the parameters and design details of MINN. Now, we will verify the effectiveness of MINN in solving the Sylvester equation problem. First, let $\tau(t) = 5$ and

$$\boldsymbol{\alpha} = \begin{bmatrix} -4 & 0.3 & 0.2 & 0.5 \\ 0.3 & -4 & 0.1 & 0.6 \\ 0.2 & 0.1 & -4 & 0.4 \\ 0.5 & 0.6 & 0.4 & -4 \end{bmatrix}$$

then, Supplementary Fig. 1 shows the state process of MINN under four activation functions. The blue curve represents the system state, and the red curve represents the desired trajectory. In three experiments with different initial states, all states of the system converged quickly to the desired trajectory. The results in Supplementary Fig. 1 verify the effectiveness of the proposed MINN in solving the Sylvester equation.

Additionally, we present the residual images of MINN, VPRNN, and ZNN under different activation functions. It should be noted that all experiments in Supplementary Fig. 2 were conducted under the same conditions. It can be observed that among the three networks, MINN exhibits the best convergence performance. Furthermore, under the ReLU activation function, only MINN can achieve system convergence. Overall, the results in Supplementary Figs. 1 and 2 confirm that MINN performs well in solving the Sylvester equation problem. In terms of convergence, MINN outperforms the currently popular ZNN and VPRNN.

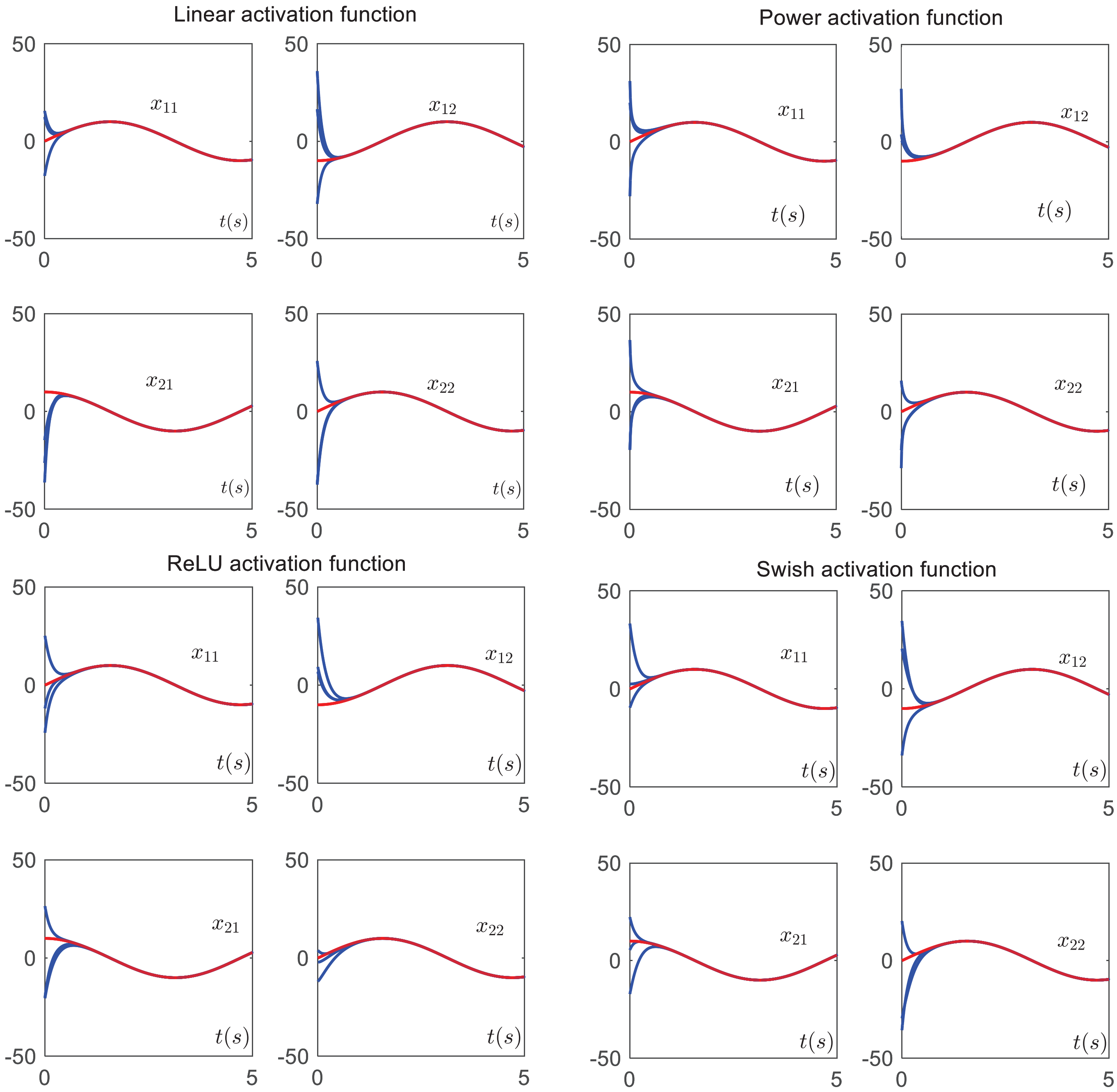

Supplementary Fig. 1 State Trajectory. The evolution of system state when solving the Sylvester equation using MINN under different activation functions (Linear, Power, ReLU, Swish). The red line represents the expected value, and the blue curve represents the state evolution curve under three different initial values.

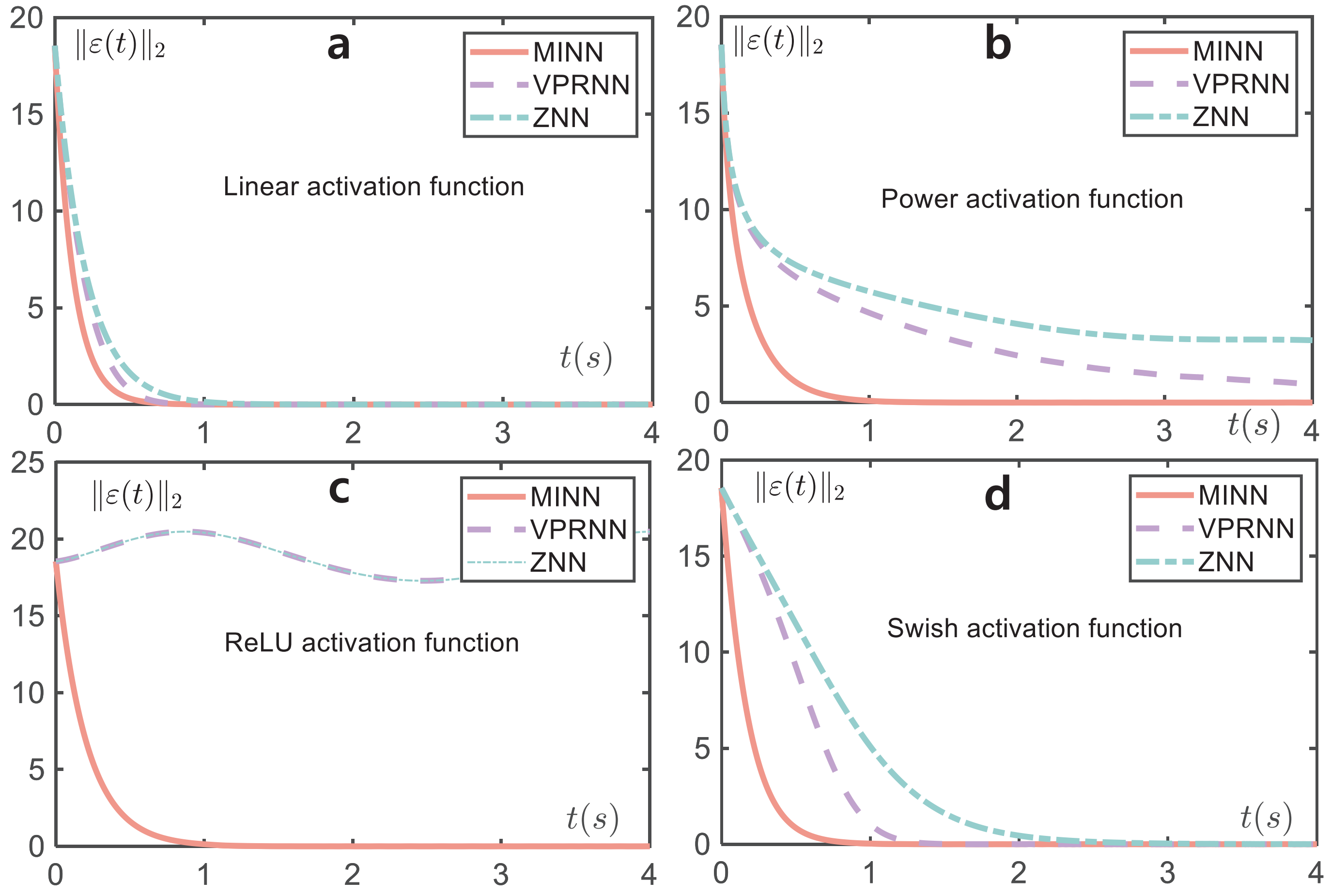

Supplementary Fig. 2 The evolution of residuals when solving the Sylvester equation using three models under different activation functions. a Linear activation function. **b** Power activation function. **c** ReLU activation function. **d** Swish activation function.

Supplementary Note 2

Robustness of MINN for solving time-varying quadratic programming problem

Ref. [3] describes a neural network model with disturbances. Thus, considering differentiation errors and model-implementation errors, a perturbed model of MINN can be described as

$$\boldsymbol{\Theta}(t)\dot{\mathbf{x}}(t) = -(\boldsymbol{\Psi}(t) + \Delta\boldsymbol{\Psi}(t))\mathbf{x}(t) - \boldsymbol{\Upsilon}(t) - \tau(t)(\boldsymbol{\Xi}(t)\mathbf{x}(t) + \boldsymbol{\Phi}(t)) + \Delta\mathbf{s}(t) + \boldsymbol{\alpha}\mathbf{F}(\boldsymbol{\Xi}(t)\mathbf{x}(t) + \boldsymbol{\Phi}(t))$$

(Supplementary Equation 5)

where $\Delta\boldsymbol{\Psi}(t) \in \mathbb{R}^{\nu\times\nu}$ denotes the differentiation error of coefficient matrix $\boldsymbol{\Psi}(t)$, and $\Delta\mathbf{s}(t) \in \mathbb{R}^{\nu}$ denotes the model-implementation error. Setting network parameters $\tau(t) = 2$, the system state is limited to [-3, 3], and

$$\Delta\boldsymbol{\Psi}(t) = \begin{bmatrix} 2cos(t) & cos(t) & sin(t) & cos(2t) & sin(t) & 2cos(t) & cos(3t) \\ cos(t) & sin(2t) & cos(t) & cos(2t) & 3sin(t) & 2sin(t) & cos(t) \\ sin(t) & cos(t) & cos(t) & sin(t) & 2cos(t) & cos(3t) & sin(t) \\ cos(2t) & cos(2t) & sin(t) & sin(t) & 2cos(t) & cos(2t) & 3cos(t) \\ sin(t) & 3sin(t) & 2cos(t) & 2cos(t) & 2cos(t) & sin(3t) & 2cos(t) \\ 2cos(t) & 2sin(t) & cos(3t) & cos(2t) & sin(3t) & sin(t) & cos(t) \\ cos(3t) & cos(t) & sin(t) & 3cos(t) & 2cos(t) & cos(t) & cos(2t) \end{bmatrix}, \Delta\mathbf{s}(t) = \begin{bmatrix} cos(2t) \\ 2cos(t) \\ sin(t) \\ 3cos(2t) \\ sin(2t) \\ 2cos(t) \\ cos(t) \end{bmatrix}$$

the other parameters are consistent with those in the experimental section of the main text. Supplementary Fig. 3 shows the residuals of ZNN, VPRNN, and MINN when solving the TVQP problem under perturbations using two activation functions. Among them, the linear activation function represents a monotonically increasing activation function type, while the Swish activation function represents a non-monotonically increasing type. Moreover, In Supplementary Fig. 3, ZNN cannot guarantee convergence of residuals under perturbations, while VPRNN can converge the system but at a slower rate than MINN. Overall, MINN exhibits the best robustness and effectively addresses the convergence issue of the TVQP problem under perturbations.

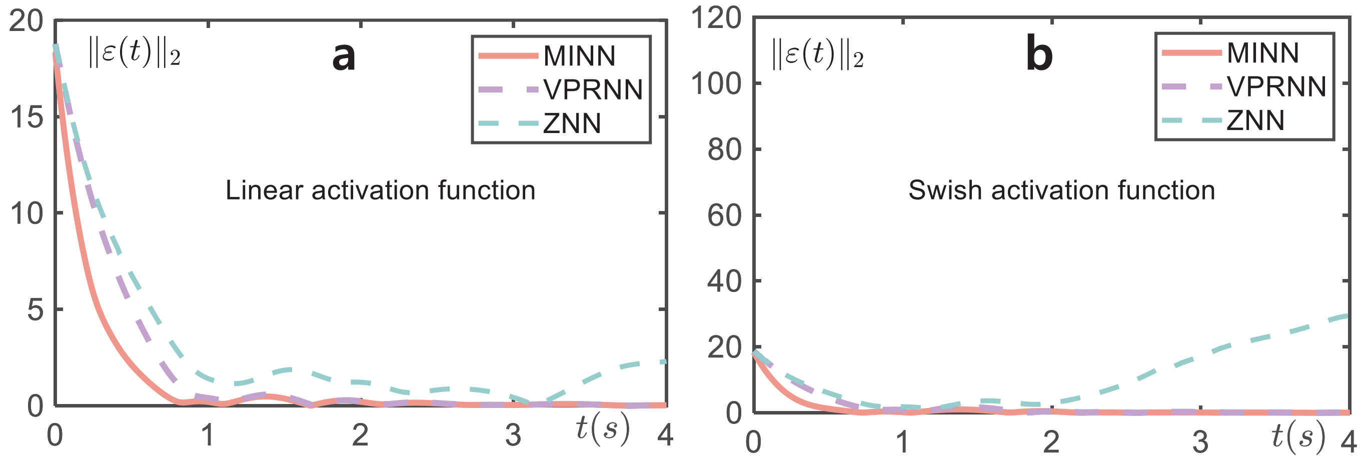

Supplementary Fig. 3 Results of the robustness. The evolution of residuals when solving TVQP with perturbations under different activation functions in three models. **a** Linear activation function. **b** Swish activation function.

Supplementary Note 3

Supplementary information for robot experiments

The motion planning problem for UFACTORY xArm 6 robot can be summarized as follows:

$$\begin{aligned} \min.\ & \dot{\boldsymbol{\theta}}^{\mathrm{T}}(t)\dot{\boldsymbol{\theta}}(t)/2 \\ \text{s.t.}\ & J(\boldsymbol{\theta}(t))(\dot{\boldsymbol{\theta}}(t)) = \dot{\mathbf{z}}(t) \\ & \mathbf{H}\dot{\boldsymbol{\theta}}(t) \leq \mathbf{K} \end{aligned} \qquad \text{(Supplementary Equation 6)}$$

where the equality constraints in the constraint equations are the derivatives of the robot's kinematic equations with respect to time. The inequality constraints are the physical limits of the robot's joints (angles and angular velocities), and $\mathbf{H} = \begin{bmatrix} \mathbf{I} & 0 \\ 0 & -\mathbf{I} \end{bmatrix}$, $\mathbf{K} = \begin{bmatrix} \mathbf{K}^{+} \\ -\mathbf{K}^{-} \end{bmatrix}$, $\mathbf{I}$ is unit matrix. the physical limits of the joints must be taken into account in the motion planning for the manipulators. Excessive angular and angular velocity outputs can cause damage to the manipulators. Therefore the physical limits of the robot is set as

$$\boldsymbol{\theta}(t) \in [\theta^{-}, \theta^{+}], \dot{\boldsymbol{\theta}}(t) \in [\dot{\theta}^{-}, \dot{\theta}^{+}] \qquad \text{(Supplementary Equation 7)}$$

where θ_i^{-}, θ_i^{+}, $\dot{\theta}_i^{-}$ and $\dot{\theta}_i^{+}$ denote the upper and lower bounds of joint angles and angular velocities, respectively. $\boldsymbol{\theta}(t) = [\theta_1(t), \theta_2(t), \cdots, \theta_n(t)]^{\mathrm{T}}$ is the joint vector for the n-dimensional robot manipulator.

According to [4], the constraints on the joints is solved at the velocity level. Thus, (Supplementary Equation 7) is rewritten as

$$\varrho^{-} = \varrho(\theta^{-} - \theta) \leq \dot{\boldsymbol{\theta}} \leq \varrho(\theta^{+} - \theta) = \varrho^{+}$$

where ϱ is a specific parameter of manipulator, and the bound constraint is

$$\mathbf{K}^{-} = \max\{\varrho_i^{-}, \dot{\theta}^{-}\} \leq \dot{\boldsymbol{\theta}} \leq \min\{\varrho^{+}, \dot{\theta}^{+}\} = \mathbf{K}^{+}. \qquad \text{(Supplementary Equation 8)}$$

Then, the physical limit constraints of the joints is obtain as $\mathbf{K}^{-} \leq \dot{\boldsymbol{\theta}} \leq \mathbf{K}^{+}$, where $\mathbf{K}^{-} = [K_1^{-\mathrm{T}}, K_2^{-\mathrm{T}}, \ldots, K_n^{-\mathrm{T}}]^{\mathrm{T}}$ and $\mathbf{K}^{+} = [K_1^{+\mathrm{T}}, K_2^{+\mathrm{T}}, \ldots, K_n^{+\mathrm{T}}]^{\mathrm{T}}$. Furthermore, the physical limit constraints on the joints can be summarized as $\mathbf{H}\dot{\theta} \leq \mathbf{K}$.

Now, we set the initial joint angle of the robotic arm as [0;-2.3047;0.8002;0;-0.3473;0]rad, and all joint angles and angular velocity limits are 2.5rad and 1.2rad/s, respectively. Then, Supplementary Fig. 4 shows the evolution of the angles and angular velocities of each joint of the robot under MINN over time. It can be seen that the physical limits of the robot's joints are restricted to a predetermined range, and when the angular velocity reaches the threshold, it will remain within but not exceed the set limit value.

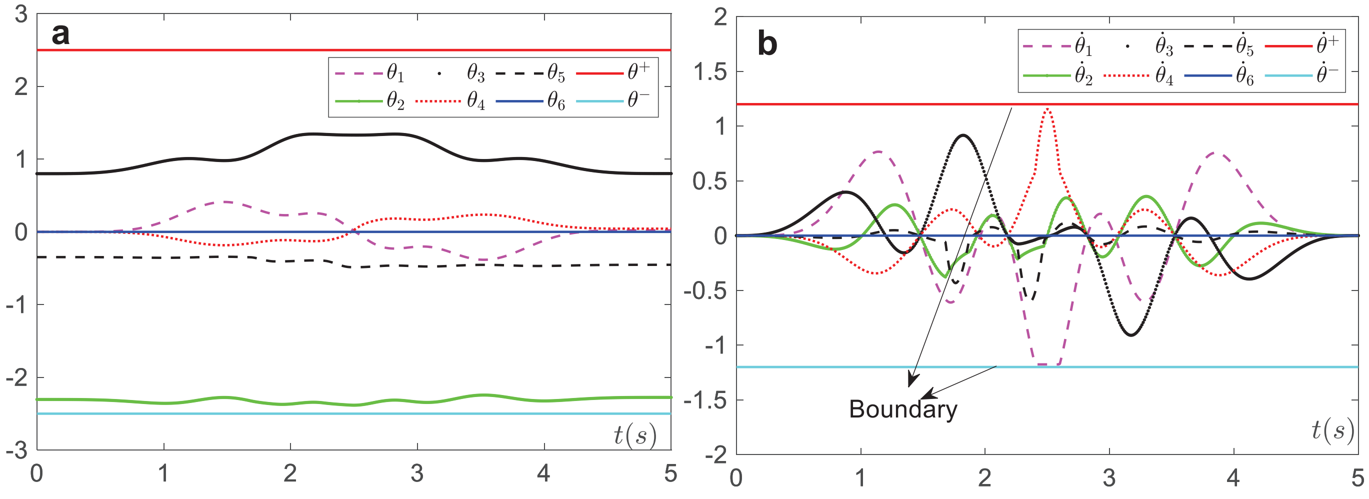

Supplementary Fig. 4 Status of the robot joint. Evolution of joint angles and angular velocities when solving robot motion planning using MINN. **a** joint angles. **b** angular velocities.

Supplementary Note 4

Necessary Lemmas and Mathematical Transformations

For TVQP problem

$$\begin{aligned} \text{min.} \quad & \mathbf{m}^{\mathrm{T}}(t)\mathbf{A}(t)\mathbf{m}(t)/2 + \mathbf{B}^{\mathrm{T}}(t)\mathbf{m}(t) \\ \text{s. t.} \quad & \mathbf{C}(t)\mathbf{m}(t) = \mathbf{D}(t) \\ & \mathbf{P}(t)\mathbf{m}(t) \leq \mathbf{Q}(t) \end{aligned} \qquad \text{(Supplementary Equation 9)}$$

ref. [5] established that the following KKT conditions guarantee the existence of an optimal solution, that is

Lemma 1: (KKT condition [5]) If and only if there exist Lagrangian multipliers $\boldsymbol{\lambda}^*(t) \in \mathbb{R}^u$ and $\boldsymbol{\mu}^*(t) \in \mathbb{R}^l$ such that

$$\begin{cases} \mathbf{A}(t)\mathbf{m}^*(t) + \mathbf{B}(t) + \mathbf{C}^{\mathrm{T}}(t)\boldsymbol{\lambda}^*(t) + \mathbf{P}^{\mathrm{T}}(t)\boldsymbol{\mu}^*(t) = 0 \\ \mathbf{C}(t)\mathbf{m}^*(t) - \mathbf{D}(t) = 0 \\ \boldsymbol{\mu}^*(t) \geq 0 \\ \mathbf{Q}(t) - \mathbf{P}(t)\mathbf{m}^*(t) \geq 0 \\ \boldsymbol{\mu}^{*\mathrm{T}}(t)(\mathbf{Q}(t) - \mathbf{P}(t)\mathbf{m}^*(t)) = 0 \end{cases} \qquad \text{(Supplementary Equation 10)}$$

where $\mathbf{m}^*(t)$ is optimal solution for TVQP problem. Moreover, if $\mathbf{A}(t)$ is positive-definite, $\mathbf{m}^*(t)$ is the optimal solution for (Supplementary Equation 9) if and only if $\mathbf{m}^*(t)$ is the KKT point to (Supplementary Equation 9).

The inequalities in (Supplementary Equation 10) hinder straightforward mathematical modeling. For this reason, the nonlinear complementary function is employed [6], i.e.,

$$\aleph(\mathbf{Q}(t) - \mathbf{P}(t)\mathbf{m}^*(t), \mu^*(t)) = 0 \Longleftrightarrow \begin{cases} \mathbf{Q}(t) - \mathbf{P}(t)\mathbf{m}^*(t) \geq 0 \\ \boldsymbol{\mu}^*(t) \geq 0 \\ (\mathbf{Q}(t) - \mathbf{P}(t)\mathbf{m}^*(t)) \circ \boldsymbol{\mu}^*(t) = 0 \end{cases}$$

where $\circ$ is Hadamard product. Let $\boldsymbol{\phi}(t) = \mathbf{Q}(t) - \mathbf{P}(t)\mathbf{m}^*(t)$, and using the perturbed Fischer-Burmeister (FB) function [6] as the nonlinear complementary problem function, i.e.,

$$\boldsymbol{\aleph}_{\mathrm{FB}}^{\delta}(\boldsymbol{\phi}(t), \boldsymbol{\mu}^*(t)) = \boldsymbol{\phi}(t) + \boldsymbol{\mu}^*(t) - \sqrt{\boldsymbol{\phi}(t) \circ \boldsymbol{\phi}(t) + \boldsymbol{\mu}^*(t) \circ \boldsymbol{\mu}^*(t) + \delta} \qquad \text{(Supplementary Equation 11)}$$

where $\delta \to 0_+$ is a small constant.

Supplementary Note 5

Proof of Theorem 1

Proof: Choosing the Lyapunov functional candidate as

$$V(t) = \|\boldsymbol{\varepsilon}(t)\|_2^2/2 =\geq 0. \qquad \text{(Supplementary Equation 12)}$$

The derivative of (Supplementary Equation 12) along time t is

$$\begin{aligned}
\dot{\boldsymbol{V}}(t) = \boldsymbol{\varepsilon}^T(t)\frac{d\boldsymbol{\varepsilon}(t)}{dt} &= -\tau(t)\boldsymbol{\varepsilon}^T(t)\boldsymbol{\varepsilon}(t) + \boldsymbol{\alpha}\boldsymbol{\varepsilon}^T(t)\mathbf{F}(\boldsymbol{\varepsilon}(t)) \\
&= -\tau(t)\sum_{i=1}^{\nu}\varepsilon_i^2 + \sum_{i=1}^{\nu}\sum_{j=1}^{\nu}\alpha_{ij}\varepsilon_i(t)f_j(\varepsilon_j(t)) \\
&= -\tau(t)\sum_{i=1}^{\nu}\varepsilon_i^2 + \sum_{i=1}^{\nu}\sum_{j=1}^{\nu}\alpha_{ij}\varsigma_i(t)\varepsilon_i(t)\varepsilon_j(t) \\
&\leq -\tau(t)\sum_{i=1}^{\nu}\varepsilon_i^2 + \sum_{i=1}^{\nu}\sum_{j=1}^{\nu}\frac{\alpha_{ij}\varsigma_j(t)}{2}(\varepsilon_i^2(t) + \varepsilon_j^2(t)) \\
&= \sum_{i=1}^{\nu}\left\{[-\tau(t) + \sum_{j=1}^{\nu}\frac{\alpha_{ij}\varsigma_j(t)}{2}]\varepsilon_i^2 + \sum_{j=1}^{\nu}\frac{\alpha_{ij}\varsigma_j(t)\varepsilon_j^2(t)}{2}\right\}
\end{aligned} \qquad \text{(Supplementary Equation 13)}$$

if the activation function $\mathbf{F}(\cdot)$ satisfies Theorem 1, then, according to $\sum_{j=1}^{\nu}\alpha_{ij} \leq 0$ and $\varsigma_i(t) > 0$ we have $\dot{V}(t) \leq 0$, which means that $\lim_{t\to+\infty}\mathbf{x}^*(t) - \mathbf{x}(t) = 0$.

To further illustrate the accuracy of (Supplementary Equation 13), the four activation functions that satisfy Theorem 1 are chosen as

$$f_i(\varepsilon_i(t)) = \begin{cases} \varepsilon_i(t), & \text{Linear,} \\ \varepsilon_i^{\gamma}(t), \gamma > 1 & \text{Power,} \\ \frac{\varepsilon_i(t)}{1+e^{-\varepsilon_i(t)}} & \text{Swish,} \\ \max(0, \varepsilon_i(t)) & \text{ReLU,} \end{cases} \qquad \text{(Supplementary Equation 14)}$$

where γ is an odd number.

Case 1 Linear. Combining Eqs.(5) and (10) in main text, we have

$$\begin{aligned}
\dot{V}_{\text{Linear}}(t) =& \boldsymbol{\varepsilon}^T(t)\frac{d\boldsymbol{\varepsilon}(t)}{dt} \\
=& \varepsilon_1(t)\dot{\varepsilon}_1(t) + \varepsilon_2(t)\dot{\varepsilon}_2(t) + \cdots + \varepsilon_\nu(t)\dot{\varepsilon}_\nu(t) \\
=& -\tau(t)\varepsilon_1^2(t) + \alpha_{11}\varepsilon_1^2(t) + \alpha_{12}\varepsilon_1(t)\varepsilon_2(t) + \cdots + \alpha_{1\nu}\varepsilon_1(t)\varepsilon_\nu(t) \\
& -\tau(t)\varepsilon_2^2(t) + \alpha_{21}\varepsilon_2(t)\varepsilon_1(t) + \alpha_{22}\varepsilon_2^2(t) + \cdots + \alpha_{2\nu}\varepsilon_2(t)\varepsilon_\nu(t) \\
& \cdots \\
& -\tau(t)\varepsilon_\nu^2(t) + \alpha_{\nu1}\varepsilon_\nu(t)\varepsilon_1(t) + \alpha_{\nu2}\varepsilon_\nu(t)\varepsilon_2(t) + \cdots + \alpha_{\nu\nu}(t)\varepsilon_\nu^2(t).
\end{aligned} \qquad \text{(Supplementary Equation 15)}$$

Under the assumption of topological symmetry, i.e. $\alpha_{ij} = \alpha_{ji}$. Then, after simple inequality techniques and

mathematical conversions, (Supplementary Equation 15) can be rewritten as

$$\begin{aligned}\dot{V}_{\text{Linear}}(t) \leq & [-\tau(t) + \alpha_{11} + \alpha_{12} + \cdots + \alpha_{1\nu}]\varepsilon_1^2(t) \\ & + [-\tau(t) + \alpha_{21} + \alpha_{22} + \cdots + \alpha_{2\nu}]\varepsilon_2^2(t) \\ & \cdots \\ & + [-\tau(t) + \alpha_{\nu 1} + \alpha_{\nu 2} + \cdots + \alpha_{\nu\nu}]\varepsilon_\nu^2(t)\end{aligned} \quad \text{(Supplementary Equation 16)}$$

since $\tau(t) > 0$, α is a diagonally dominant matrix, we have $\sum_{j=1}^{\nu} \alpha_{ij} < 0$ and $\dot{V}_{\text{Linear}}(t) \leq 0$.

Case 2 Power. Similar to the derivation of (Supplementary Equation 15) and (Supplementary Equation 16), the derivative of (9) in main text along time under the Power activation function is directly given as

$$\begin{aligned}\dot{V}_{\text{Power}}(t) \leq & -\tau(t)\varepsilon_1^2(t) + [\alpha_{11}\varepsilon_1^2(t) + \alpha_{12} + \cdots + \alpha_{1\nu}]\varepsilon_1^{\gamma+1}(t) \\ & -\tau(t)\varepsilon_2^2(t) + [\alpha_{21} + \alpha_{22}\varepsilon_2^2(t) + \cdots + \alpha_{2\nu}]\varepsilon_2^{\gamma+1}(t) \\ & \cdots \\ & -\tau(t)\varepsilon_2^2(t) + [\alpha_{\nu 1} + \alpha_{\nu 2} + \cdots + \alpha_{\nu\nu}\varepsilon_\nu^2(t)]\varepsilon_\nu^{\gamma+1}(t)\end{aligned} \quad \text{(Supplementary Equation 17)}$$

since γ is an odd number and $\gamma > 1$, thus, one has $\varepsilon_i^{\gamma+1}(t) \geq 0$. Furthermore, the diagonally dominant matrix $\boldsymbol{\alpha}$ ensures that $\alpha_{i1} + \alpha_{i2} + \cdots + \alpha_{ij} \leq 0$. In summary, we have $\dot{V}_{\text{Power}}(t) \leq 0$.

Case 3 Swish. Combining the Swish activation function in (Supplementary Equation 14) and using the method in Case 1 yields

$$\begin{aligned}\dot{V}_{\text{Swish}}(t) \leq & [-\tau(t) + \frac{\alpha_{11}}{1+e^{-\varepsilon_1}} + \frac{\alpha_{12}}{1+e^{-\varepsilon_2}} + \cdots + \frac{\alpha_{1\nu}}{1+e^{-\varepsilon_\nu}}]\varepsilon_1^2(t) \\ & + [-\tau(t) + \frac{\alpha_{21}}{1+e^{-\varepsilon_1}} + \frac{\alpha_{22}}{1+e^{-\varepsilon_2}} + \cdots + \frac{\alpha_{2\nu}}{1+e^{-\varepsilon_\nu}}]\varepsilon_2^2(t) \\ & \cdots \\ & + [-\tau(t) + \frac{\alpha_{\nu 1}}{1+e^{-\varepsilon_1}} + \frac{\alpha_{\nu 2}}{1+e^{-\varepsilon_2}} + \cdots + \frac{\alpha_{\nu\nu}}{1+e^{-\varepsilon_\nu}}]\varepsilon_\nu^2(t)\end{aligned} \quad \text{(Supplementary Equation 18)}$$

since $\tau(t) > 0$ and $\sum_{j=1}^{\nu} \alpha_{ij} < 0$, therefore, $-\tau(t) + \sum_{j=1}^{\nu} \frac{\alpha_{ij}}{1+e^{-\varepsilon_j}} < 0$. Furthermore, we have $\dot{V}_{\text{Swish}}(t) \leq 0$.

Case 4 ReLU. When the error function $\boldsymbol{\varepsilon}(t) < 0$, the $\mathbf{F}(\boldsymbol{\varepsilon}(t)) = 0$. At this point, $\dot{V}_{\text{ReLU}}(t) = \boldsymbol{\varepsilon}^T(t)\frac{d\boldsymbol{\varepsilon}(t)}{dt} = -\tau(t)\varepsilon_1^2(t) - \tau(t)\varepsilon_2^2(t) - \cdots - \tau(t)\varepsilon_\nu^2(t) \leq 0$. When the error function $\boldsymbol{\varepsilon}(t) \geq 0$, the $\mathbf{F}(\boldsymbol{\varepsilon}(t)) = \boldsymbol{\varepsilon}(t)$, in other words, the ReLU activation function is equivalent to the Linear activation function. According to the proof in Case 1, it is known that $\dot{V}_{\text{ReLU}}(t) \leq 0$. Overall, $\dot{V}_{\text{ReLU}}(t) \leq 0$ for $\boldsymbol{\varepsilon}(t) \in (-\infty, +\infty)$. The proof is finished. ■

Supplementary Note 6

Proof of Theorem 2

Proof: It has been stated in detail that VPRNN has better convergence than ZNN [7]. Therefore, it is only necessary to show that the proposed MINN has faster convergence than VPRNN in this proof. Moreover, since $V(t)$ in (Supplementary Equation 12) denotes the square of the error function, it is sufficient to show that the convergence of the MINN is superior to that of the VPRNN by proving that $\dot{V}_{\text{MINN}}(t) < \dot{V}_{\text{VPRNN}}(t) \leq 0$. To do so, define the design equation for VPRNN as

$$\dot{\boldsymbol{\varepsilon}}(t) = -\tau(t)\mathbf{F}(\boldsymbol{\varepsilon}(t)) \qquad \text{(Supplementary Equation 19)}$$

and according to (11) in main text and (Supplementary Equation 12), $\dot{V}_{\text{VPRNN}}(t)$ for (Supplementary Equation 19) can be written as

$$\dot{V}_{\text{VPRNN}}(t) = \boldsymbol{\varepsilon}^T(t)\frac{d\boldsymbol{\varepsilon}(t)}{dt} = -\tau(t)\boldsymbol{\varepsilon}^T(t)\mathbf{F}(\boldsymbol{\varepsilon}(t)) = -\sum_{i=1}^{\nu}\tau(t)\varsigma_i(t)\varepsilon_i^2(t). \qquad \text{(Supplementary Equation 20)}$$

Furthermore, combining (Supplementary Equation 13) and (Supplementary Equation 20) yields

$$\begin{aligned}
&\dot{V}_{\text{MINN}}(t) - \dot{V}_{\text{VPRNN}}(t) \\
&= -\tau(t)\sum_{i=1}^{\nu}\varepsilon_i^2 + \sum_{i=1}^{\nu}\sum_{j=1}^{v}\alpha_{ij}\varsigma_j(t)\varepsilon_i(t)\varepsilon_j(t) + \sum_{i=1}^{\nu}\tau(t)\varsigma_i(t)\varepsilon_i^2 \\
&= -\tau(t)\sum_{i=1}^{\nu}(1-\varsigma_i(t))\varepsilon_i^2 + \sum_{i=1}^{\nu}\sum_{j=1}^{\nu}\alpha_{ij}\varsigma_j(t)\varepsilon_i(t)\varepsilon_j(t) \\
&\leq \sum_{i=1}^{\nu}\{[-\tau(t)(1-\varsigma_i(t)) + \sum_{j=1}^{\nu}\frac{\alpha_{ij}\varsigma_j(t)}{2}]\varepsilon_i^2 + \sum_{j=1}^{\nu}\frac{\alpha_{ij}\varsigma_j(t)\varepsilon_j^2(t)}{2}\}
\end{aligned} \qquad \text{(Supplementary Equation 21)}$$

now, we known that $\tau(t) > 0$ and $\sum_{j=1}^{\nu}\alpha_{ij} \leq 0$, and next we need to discuss $\varsigma_i(t)$ in for different activation function. Combining (Supplementary Equation 14), we have

$$\varsigma_i(t) = \begin{cases} 1, & \text{Linear,} \\ \varepsilon_i^{\gamma-1}(t) > 0, & \text{Power,} \\ \frac{1}{1+e^{-\varepsilon_i(t)}} \in (0,1] & \text{Swish,} \\ 0 \text{ or } 1 & \text{ReLU,} \end{cases}$$

for Linear, Swish and ReLU activation functions there are $\varsigma_i(t) \in (0,1]$, thus, $1-\varsigma_i(t) \leq 0$, and one can easily obtain $\dot{V}_{\text{MINN}}(t) - \dot{V}_{\text{VPRNN}}(t) \leq 0$, which indicates that the convergence rate of MINN is consistently batter than that of VPRNN.

For Power activation function, the error function $\varepsilon_i(t)$ is larger in the early stage, thus, there is a situation where $\dot{V}_{\text{MINN}}(t) - \dot{V}_{\text{VPRNN}}(t) > 0$. At this time, the convergence rate of VPRNN will be faster than that of MINN. However, as the error decreases, $1-\varsigma_i(t)$ eventually becomes negative, which means $\dot{V}_{\text{MINN}}(t) - \dot{V}_{\text{VPRNN}}(t) < 0$, the convergence rate of MINN is faster than that of VPRNN and is maintained until until the error converges to 0. Overall, MINN is more efficient than VPRNN. A detailed discussion can be found in the simulation section. This completes the proof. ∎

Supplementary References

1. Zhang, Zhijun, et al. “A new varying-parameter recurrent neural-network for online solution of time-varying Sylvester equation.” IEEE Transactions on Cybernetics 48.11 (2018): 3135-3148.
2. Gerontitis, Dimitrios, and Panagiotis Tzekis. “Solving the generalized Sylvester equation with a novel fast extended neurodynamics.” Numerical Algebra, Control and Optimization 15.3 (2025): 619-644.
3. Zhang, Zhijun, et al. “A new varying-parameter convergent-differential neural-network for solving time-varying convex QP problem constrained by linear-equality.” IEEE Transactions on Automatic Control 63.12 (2018): 4110-4125.
4. Ren, Xiaohui, et al. “Bicriteria velocity minimization approach of self-motion for redundant robot manipulators with varying-gain recurrent neural network.” IEEE Transactions on Cognitive and Developmental Systems 14.2 (2021): 578-587.
5. Bazaraa, Mokhtar S., Hanif D. Sherali, and Chitharanjan M. Shetty. Nonlinear programming: theory and algorithms. John wiley and sons, 2006.
6. Effati, Sohrab, and A. R. Nazemi. “Neural network models and its application for solving linear and quadratic programming problems.” Applied mathematics and Computation 172.1 (2006): 305-331.
7. Zhang, Zhijun, et al. “A new varying-parameter convergent-differential neural-network for solving time-varying convex QP problem constrained by linear-equality.” IEEE Transactions on Automatic Control 63.12 (2018): 4110-4125.
